# Activation of ryanodine-sensitive calcium store drives pseudo-allergic dermatitis *via* Mas-related G protein-coupled receptor X2 in mast cells

**DOI:** 10.3389/fimmu.2023.1207249

**Published:** 2023-06-19

**Authors:** Zhao Wang, Xi Zhao, Hongmei Zhou, Delu Che, Xiaojie Du, Dan Ye, Weihui Zeng, Songmei Geng

**Affiliations:** Department of Dermatology, The Second Affiliated Hospital of Xi’an Jiaotong University, Xi’an, China

**Keywords:** pseudo-allergy, mast cell, degranulation, MRGPRX2, ryanodine receptor, calcium

## Abstract

Mast cell (MC) activation is implicated in the pathogenesis of multiple immunodysregulatory skin disorders. Activation of an IgE-independent pseudo-allergic route has been recently found to be mainly mediated *via* Mas-Related G protein-coupled receptor X2 (MRGPRX2). Ryanodine receptor (RYR) regulates intracellular calcium liberation. Calcium mobilization is critical in the regulation of MC functional programs. However, the role of RYR in MRGPRX2-mediated pseudo-allergic skin reaction has not been fully addressed. To study the role of RYR *in vivo*, we established a murine skin pseudo-allergic reaction model. RYR inhibitor attenuated MRGPRX2 ligand substance P (SP)-induced vascular permeability and neutrophil recruitment. Then, we confirmed the role of RYR in an MC line (LAD2 cells) and primary human skin-derived MCs. In LAD2 cells, RYR inhibitor pretreatment dampened MC degranulation (detected by β-hexosaminidase retlease), calcium mobilization, IL-13, TNF-α, CCL-1, CCL-2 mRNA, and protein expression activated by MRGPRX2 ligands, namely, compound 48/80 (c48/80) and SP. Moreover, the inhibition effect of c48/80 by RYR inhibitor was verified in skin MCs. After the confirmation of RYR2 and RYR3 expression, the isoforms were silenced by siRNA-mediated knockdown. MRGPRX2-induced LAD2 cell exocytosis and cytokine generation were substantially inhibited by RYR3 knockdown, while RYR2 had less contribution. Collectively, our finding suggests that RYR activation contributes to MRGPRX2-triggered pseudo-allergic dermatitis, and provides a potential approach for MRGPRX2-mediated disorders.

## 1 Introduction

Skin hypersensitivity reaction is caused by skin exposure to allergens. Mast cells are regarded as the primary cells that contributes to this process (1–3). Mast cell activation is implicated in a wide spectrum of dermatoses including chronic allergic contact dermatitis and atopic dermatitis (4). Although mast cell activation has been investigated for decades, the primary trigger is believed to be IgE. However, not all patients respond to anti-IgE treatment (5, 6). Moreover, MC secretagogues such as compound 48/80 (c48/80) and neuropeptide substance P (SP) have been applied to induce experimental itch in pseudo-allergic patients and MCs *ex vivo* for decades (7, 8), which does not rely on the IgE-dependent route and was termed pseudo-allergic reaction. Meanwhile, SP was positively correlated with disease severity (9, 10). This indicates the existence of an alternative pathway.

In recent years, Mas-related G protein-coupled receptor X2 (MRGPRX2) was identified to be expressed on skin mast cells and basophils (mouse orthologue is MrgprB2) (11, 12). MRGPRX2 mediates a type of allergic reaction which resembles the symptom of IgE-triggered hypersensitivity reactions (13). The finding of MRGPRX2 explains the phenomenon that patients exhibited skin allergic responses while serum IgE was not elevated. Although MRGPRX2 is deeply mediated in multiple pseudo-allergic skin reactions and is implicated in several skin diseases, the mechanism of MRGPRX2-mediated MC activation is under investigation.

Calcium signaling is crucial for mast cell activation and is implicated in both MC degranulation and the generation of cytokines. Calcium activation patterns by MRGPRX2 and FcϵRI are drastically diverse (14, 15). MRGPRX2-triggered calcium influx is rapid, while FcϵRI-mediated calcium activation is relatively delayed. Multiple calcium channels are expressed on human skin MC. Intracellular and extracellular calcium synergistically regulate cellular events, which are regulated by the activation of diverse calcium channels (16). Intracellular calcium release comes from the activation of calcium channels expressed on the sarco/endoplasmic reticulum and mitochondria (16). Inositol 1,4,5-trisphosphate receptor (IP3R) activation-induced intracellular calcium mobilization has been confirmed in both FcϵRI- and MRGPRX2-mediated routes (17, 18).

Ryanodine receptor (RYR) is one of the primary intracellular calcium release channels apart from IP3R. RYR is known for regulating the contraction of muscles and widely expresses in the sarco/endoplasmic reticulum (19, 20). Ryanodine is a plant component, which has high affinity to RYR. It has divergent roles depending on the concentration, and nanomole concentrations activate RYR by holding it in an open subconductance state, while a higher concentration inhibits the channel (21). Previous publications employed ryanodine as an intracellular depletor (22, 23). With the application of RYR inhibitor, namely, dantrolene, RYR can be more specifically targeted (24). In rat peritoneal mast cells, dantrolene inhibited calcium mobilization and histamine release activated by FcϵRI aggregation (25). However, it remains unknown if the activation of MRGPRX2 triggered functional regulation of MC relies on RYR-sensitive calcium signals. Thus, in the present study, we aimed to elucidate the mechanism of calcium signaling by RYR activation, which is elicited by MRGPRX2 activation.

## 2 Materials and methods

### 2.1 Mice

C57BL/6 mice (8–10 weeks of age, weighing 20–25 g, male and female) were obtained from the Laboratory Animal Center of Xi’an Jiaotong University. Animal experiments were approved by the Ethics Committee of the Second Affiliated Hospital of Xi’an Jiaotong University (protocol code 2022125, 7 June 2022). Animals were housed under standard conditions (20–25°C, relative humidity 40%, light and dark cycle 12 h) with free access to water and standard dry feed. Mice were randomly divided into a control group and an experimental group.

### 2.2 Murine Evans blue dye extravasation model

C57BL/6 mice (8–10 weeks of age, weighing 20–25 g, male and female) were intraperitoneally injected with 50 µl of dantrolene (10 mg/kg in DMSO) or vehicle for 2 consecutive days. Then, each mouse was injected intravenously with 0.2 ml of 0.4% Evans blue dye in saline. After 1 h, mice were anesthetized with an intraperitoneal injection of 80 mg/kg 1% pelltobarbitalum natricum. The thickness of the ear was determined by a vernier caliper. Then, an intradermal injection of SP (50 µM in 20 µl saline) was performed randomly into one side of the ear, and the vehicle (saline) was intradermally injected into the other. After 30 min, the mice were euthanized, ear thickness was measured again, and pictures of the ears were taken. Ear tissues were collected, dried for 24 h at 60°C, and weighed. Tissues were immersed and minced in 300 μl of acetone-saline (7:3). Following by 30 min ultrasonication, tissues were centrifuged for 20 min at 3,000 rpm. Two-hundred-microliter aliquots of the supernatant were seeded into 96-well plates, and the optical density (OD) was read at 620 nm.

### 2.3 *In vivo* murine skin pseudo-allergic reaction model

C57BL/6 mice (8–10 weeks of age, weighing 20–25 g, male and female) were intraperitoneally injected with 50 µl of dantrolene (10 mg/kg in DMSO) or saline of the same volume for 2 consecutive days. SP (50 µl of 200 µM SP in saline) or vehicle were intradermally injected into one side of the ear pinna to activate MrgprB2 (murine orthologue of human MRGPRX2). Three hours later, all mice were euthanized and ears were cut and digested with 1.5 mg/ml collagenase type IV (Gibco, Waltham, USA) with DNase I at 10 μg/ml (Roche, Basel, Switzerland) at 37°C in a shaking incubator for 75 min. Cells were filtrated and centrifuged at 400×*g* for 10 min at 4°C. Pellet-containing cells were processed for flow cytometric analysis.

### 2.4 Flow cytometry

Cells were incubated with CD11b-PE (Clone:M1/70) and Ly6G-APC (Clone: RB6-8C5) for 45 min, all from liankebio (Hangzhou, China). Live versus dead cells were stained using Zombie Yellow™ Fixable viability dye (BioLegend, CA). The data were acquired with a BD FACSCelesta flow cytometer (San Jose, CA) and analyzed by FlowJo software version 10.7.2 (Tree Star Inc., Ashland, OR). Neutrophils were gated as CD11b+Ly6G+ live cells.

### 2.5 Cell line cultures

The human mast cell Laboratory Allergic Disease 2 (LAD2) cell line was kindly provided by A. Kirshenbaum and D. Metcalfe (NIH, USA). LAD2 cells were cultured with Basal Iscove’s medium (Procell Life Science & Technology Co., Ltd., Wuhan, China) supplemented with 10% FCS (VivaCell, Shanghai, China) at 37°C with 5% CO_2_. Hemidepletion was performed once a week and supplements of SCF (at 100 ng/ml) (SinoBiological, Beijing, China) and IL-4 (at 20 ng/ml) (Sigma-Aldrich, California, USA) were provided once a week.

### 2.6 Human skin mast cell isolation

Foreskins were obtained from circumcisions with the written informed consent of the patients or their legal guardians. The study was approved by the Ethics Committee of the Second Affiliated Hospital of Xi’an Jiaotong University (protocol code 2022125, 7 June 2022) and experiments were conducted according to the Declaration of Helsinki Principles. Briefly, skin samples were cut into strips and digested with a 3.5 U/ml dispase (Sigma-Aldrich, California, USA) at 4°C overnight to remove the epidermis. Then, the dermis was chopped and digested with 1.5 mg/ml collagenase type 1 (Gibco, Waltham, USA) and 0.75 mg/ml hyaluronidase type 1-S (Sigma-Aldrich, California, USA) with DNase I at 10 μg/ml (Roche, Basel, Switzerland) at 37°C in a shaking incubator for 75 min. Cells were filtrated and labeled with anti-human c-Kit magnetic microbeads to positively select MC by cell preparation columns (both from Miltenyi Biotec, Bergisch Gladbach, Germany). Purified skin MCs were cultured in Basal Iscove’s medium with 10% FCS at the concentration of 5 × 10^5^/ml, supplemented with SCF (at 100 ng/ml) and IL-4 (at 20 ng/ml) twice a week.

### 2.7 β-Hexosaminidase release assay

Cells (5 × 10^4^) treated with or without RYR inhibitor (dantrolene, 100 µM, MedChemExpress, Monmouth Junction, USA) were resuspended in 100 µl of PAG-CM buffer (Piperazine-N,N-bis[2-ethanesulfonic acid]-Albumin-Glucose buffer containing 3 mM CaCl_2_ and 1.5 mM MgCl_2_, pH 7.4) and stimulated with vehicle (spontaneous release), compound 48/80 (c48/80) (5 µg/ml, Sigma-Aldrich, St. Louis, Missouri) or substance P (SP) (30 μM, MedChemExpress, Monmouth Junction, USA) for 60 min. Cells were centrifuged and supernatants (SNs) were collected and the pelleted MCs were rapidly frozen with 100 μl of H_2_O. After thawing, 50 μl of SNs or cell lysates were incubated with the same volume of 4-methyl umbelliferyl-N-acetyl-beta-D-glucosaminide (Sigma-Aldrich, Munich, Germany) solution at 5 μM in citrate buffer (pH 4.5) for 60 min at 37°C. Sodium carbonate buffer (100 mM; pH 10.7) was added to stop the reaction. Fluorescence intensity was determined at excitation at 355 nm and emission wavelength of 460 nm. % β‐hexosaminidase release = [fluorescence intensity SN/(fluorescence intensity SN + fluorescence intensity lysate)] × 100. The net release was calculated by subtracting spontaneous release.

### 2.8 Intracellular calcium mobilization assay

Cells (1 × 10^5^ cells/sample) were loaded with 2 μM Fluo-4 AM (Beyotime, Shanghai, China) with 0.02% Pluronic F-127 (Beyotime, Shanghai, China) for 45 min at 37°C in the dark, followed by de-esterification for an additional 15 min at room temperature. Then, the cells were washed and resuspended with PAG-CM buffer. For assay with inhibitor, cells were loaded with dantrolene (100 µM) for 15 min before ligand stimulation. Calcium signals were determined using a Fluorescence Spectrophotometer (BMG LABTECH, Ortenberg, Germany) with an excitation wavelength of 494 nm and an emission wavelength of 516 nm. The signal was recorded every 2 s for 2 min at baseline, and additionally 5 min after stimulation.

### 2.9 RT-qPCR

Briefly, total RNA was isolated with RNA fast200 (Feijie, Shanghai, China) and reverse‐transcribed with a SweScript All-in-One First-Strand cDNA Synthesis SuperMix for qPCR (Servicebio, Wuhan, China) as detailed by the manufacturer. PCR was carried out with the 2×Universal Blue SYBR Green qPCR Master Mix (Servicebio, Wuhan, China). Primers were 5’-TTGCGGAGCAAGAGATTCCC and 5’-GGCAGTGCCTCAGCATTTTT for CCL-1, 5’-CCCCAAGCAGAAGTGGGTTC and 5’-TTGGGTTGTGGAGTGAGTGTT for CCL-2, 5’-CTGGGCAGGTCTACTTTGGG and 5’-CTGGAGGCCCCAGTTTGAAT for TNF-α, and 5’-CATCCGCTCCTCAATCCTCT and 5’-GATGCTCCATACCATGCTGC for IL-13. The values were normalized to the housekeeping genes β-actin, cyclophilin B, and GAPDH. The primers were 5’-CTGGAACGGTGAAGGTGACA and 5’-AAGGACTTCCTCTAACAATGCA for β-actin, 5’-AAGATGTCCCTGTGCCCTAC and 5’-ATGGCAAGCATGTGGTGTTT for Cyclophilin B, and 5’-CCTCTGACTTCAACAGCGAC and 5’-TTACTCCTTGGAGGCCATGTG for GAPDH. The 2^−ΔΔCT^ method was used to calculate the relative expression levels of the target genes against three housekeeping genes, and the mean expression level of the target gene was calculated by dividing it against reference genes for the analysis.

### 2.10 ELISA

MCs were seeded at 1 × 10^6^ cells/ml and treated with or without dantrolene (100 µM) for 15 min and then the cells were stimulated with vehicle (spontaneous release), compound 48/80 (c48/80) (5 µg/ml), or substance P (SP) (30 μM) for 24 h. Then, supernatants were collected for IL-13, TNF-α, CCL-1, and CCL-2 measurements. ELISAs were performed according to the manufacturer’s instructions (all from Mlbio, Shanghai, China).

### 2.11 siRNA-mediated knockdown in LAD2 cells

MCs were plated at 1 × 10^6^/ml and treated with 80 nM of RYR2- and RYR3-targeting siRNA or non-targeting siRNA with RNAi-Mate (at 1 μg/ml, GenePharma, Shanghai, China) for 48 h, and employed for downstream analyses. The siRNA sequences were as follows: RYR2 forward: 5’-GGCUCUAAUUAGAGGAAAUTT, RYR2 reverse: 5’-AUUUCCUCUAAUUAGAGCCTT, RYR3 forward: 5’-GCAGAUCAACAUGCUGCUUTT, and RYR3 reverse: 5’-AAGCAGCAUGUUGAUCUGCTT.

### 2.12 Statistics

Statistical analyses were performed using PRISM 9.0 (GraphPad Software, La Jolla, CA, USA). For the difference between two groups with paired experimental design, comparisons were performed by the *t*-test (when data were normally distributed) or the Wilcoxon matched-pairs signed-rank test (when data were not normally distributed). For the difference between two groups with unpaired experimental design, the Mann–Whitney test was performed. For the difference between more than two groups, comparisons were performed by the RM one-way ANOVA with Dunnett’s multiple comparisons tests (when data were normally distributed) or Friedman test with Dunn’s multiple comparison test (when data were not normally distributed). When the data were compared to a fixed number, differences between groups were compared using the one-sample *t*-test (normally distributed) or Wilcoxon signed-rank test (not normally distributed). *p* < 0.05 was considered statistically significant.

## 3 Results

### 3.1 RYR inhibitor attenuates SP-induced vascular permeability and immune cell infiltration in the murine skin pseudo-allergic reaction model

**Figure 1:**
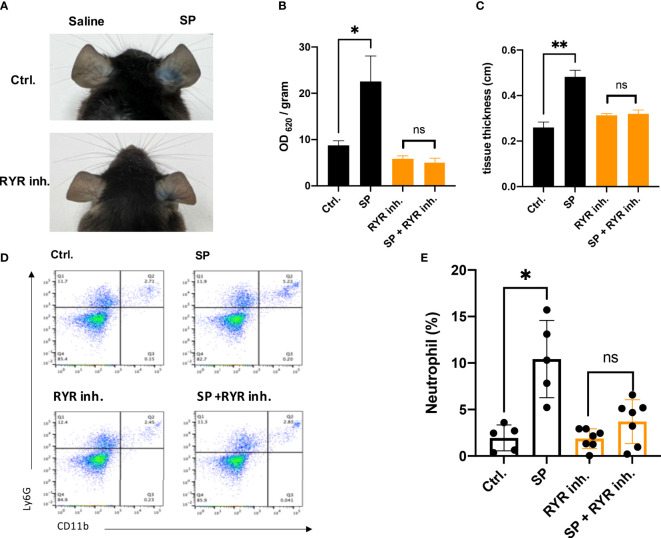
Dantrolene attenuates SP-induced *in vivo* murine skin anaphylaxis model. Dantrolene or saline was i.p. injected for 2 consecutive days, and then SP (50 µM) or saline was intradermally injected into one side of the ear. **(A–C)** Vascular permeability was quantified by Evans blue extravasation, and the thickness of the ear was determined by a vernier caliper (*n* = 6). **(A)** Representative photo of Evans blue extravasation in the murine skin anaphylaxis model. **(B)** Quantification of Evans blue extravasation. **(C)** Ear tissue thickness after the injection of SP or saline. **(D, E)** Ear tissue was collected and neutrophil infiltration (CD11b+ Ly6G+ live cells) was determined by flow cytometry (*n* = 5–7). **(D)** Representative flow cytometry images. **(E)** Percentage of neutrophils in the live skin cell population. Data shown are mean ± SEM. Ctrl.: control, inh.: inhibitor. ns: not significant, *p < 0.05, **p < 0.01.

The mouse ortholog of MRGPRX2 is MrgprB2, which resembles the activation pattern of human MRGPRX2 (11). To verify if the activation of RYR leads to the vascular permeability by MRGPRX2 activation, dantrolene was employed. Dantrolene is an inhibitor suppressing the activity of all the RYR isoforms (26, 27). We monitored Evans blue dye extravasation by intradermal injection of SP in ear pinna. For mice that received dantrolene intraperitoneally for 2 consecutive days, intradermal injection of SP leads to less Evans blue dye extravasation than that in the control group (**Figures 1A, B**). By measuring the thickness of the ear pinna, which reflects the swelling of the skin, we found that SP injection induced rapid ear swelling, yet dantrolene pre-treatment inhibited the swelling induced by SP (**Figure 1C**). Furthermore, we quantified skin immune cell infiltration by flow cytometric analysis. Neutrophil was labeled as CD11+ Ly6G+ cells. SP intradermal injection caused the recruitment of inflammatory cells in the ear pinna. Dantrolene pretreatment inhibited SP-induced neutrophil infiltration (**Figures 1D, E**).

### 3.2 RYR inhibitor perturbs MRGPRX2-mediated LAD2 degranulation and calcium mobilization

**Figure 2:**
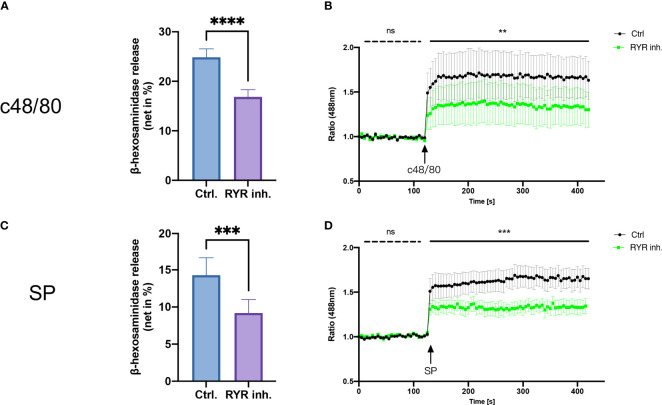
RYR inhibitor perturbs MRGPRX2-mediated mast cell degranulation and calcium mobilization. LAD2 cells were treated with RYR inhibitor (dantrolene, 100 µM) for 15 min and then the cells were stimulated with c48/80 (5 µg/ml) or SP (30 µM). **(A, B)** β‐Hexosaminidase release and **(C, D)** calcium mobilization was determined, respectively. The data are from 7 to 15 independent experiments. ns: not significant, Ctrl.: control, inh.: inhibitor. **p < 0.01, ***p < 0.001, ****p < 0.0001.

To study the role of RYR in MRGPRX2-mediated MC activation, we detected β‐hexosaminidase release in a human MC line, LAD2 cells, to assess MC degranulation. We found that dantrolene significantly inhibited both c48/80 and SP-triggered degranulation (**Figures 2A, B**). The inhibitory effect of dantrolene on c48/80-triggered degranulation was further verified in human skin-derived MCs (**Supplementary Figure 1A**).

To confirm if RYR activation regulates calcium mobilization, we detected calcium influx triggered by MRGPRX2 ligands after RYR inhibitor pre-treatment. Calcium signaling activated by MRGPRX2 ligands (both c48/80 and SP) was significantly inhibited by dantrolene (**Figures 2C, D**). Moreover, the baseline calcium signal was also inhibited by dantrolene before treatment (**Supplementary Figure 2**). Inhibited calcium mobilization by dantrolene was replicated in human skin-derived MCs (**Supplementary Figure 1B**). Thus, MRGPRX2-triggered degranulation and calcium mobilization depend on the activation of RYR.

### 3.3 MRGPRX2 activation-induced cytokine production relies on the activation of RYR

**Figure 3:**
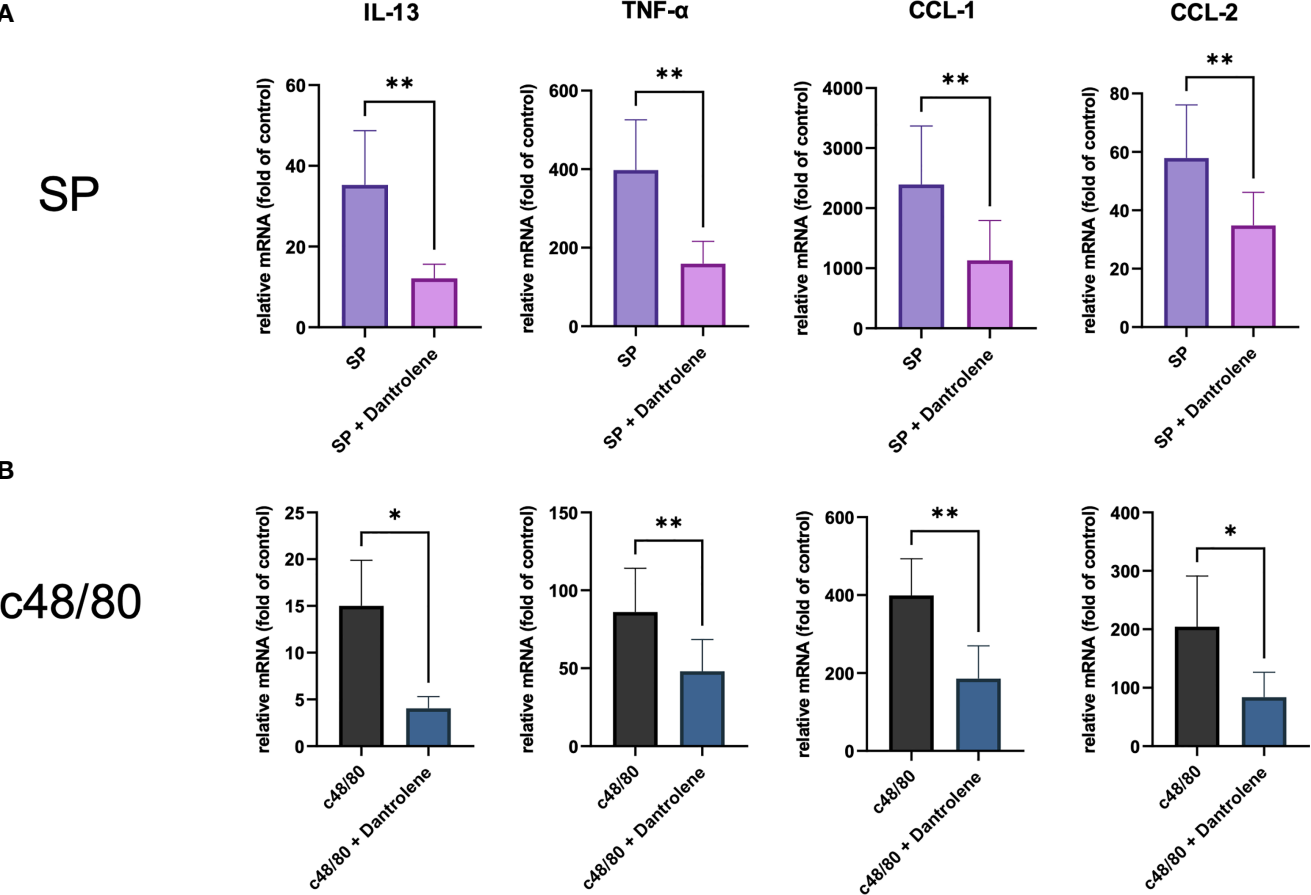
MRGPRX2 activation-induced cytokine mRNA generation relies on the activation of RYR. Cells were preincubated with or without RYR inhibitor (dantrolene, 100 µM) for 15 min and stimulated with **(A)** SP (60 µM) or **(B)** c48/80 (5 µg/ml). Cytokine mRNA was determined by RT-qPCR. The data were normalized against the cell receiving no inhibitor and stimuli. Data shown are mean ± SEM of *n* = 7–11. inh.: inhibitor. *p < 0.05, **p < 0.01.

**Figure 4:**
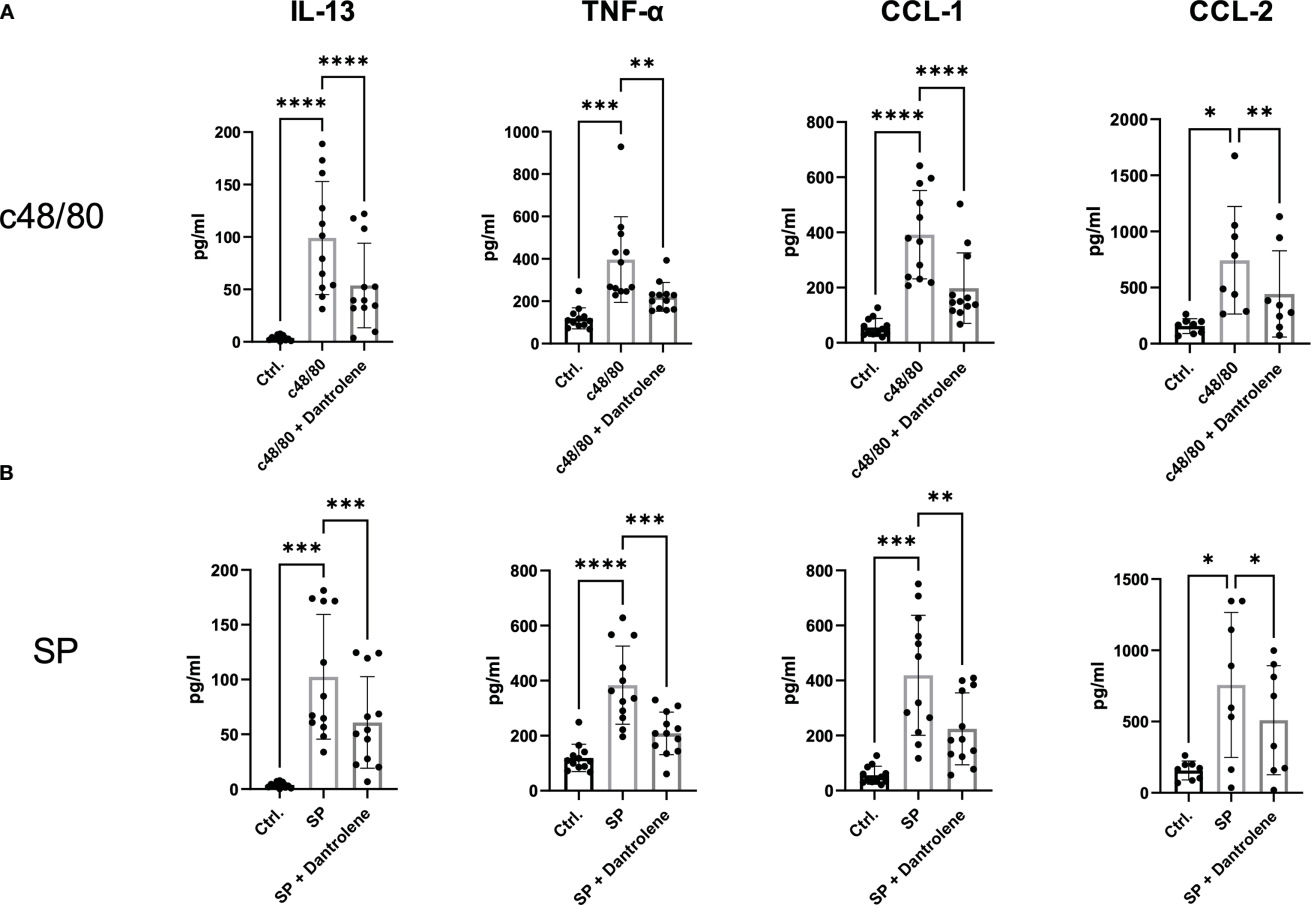
MRGPRX2-activated cytokine protein release is inhibited by RYR inhibitor. LAD2 cells were preincubated with or without RYR inhibitor (dantrolene, 100 µM) for 15 min and stimulated with **(A)** SP (60 µM) or **(B)** c48/80 (5 µg/ml) for 24 h. Supernatants were collected and IL-13, TNF-α, CCL-1, and CCL-2 protein release was quantified by ELISA. Data shown are mean ± SEM of *n* = 8–12. Ctrl.: control, inh.: inhibitor. *p < 0.05, **p < 0.01, ***p < 0.001, ****p < 0.0001.

Apart from degranulation, cytokine production is regulated by calcium signal in MCs (28–30). To further determine if RYR is implicated in MRGPRX2-triggered cytokine and chemokine generation, we detected IL-13, TNF-α, CCL-1, and CCL-2 expression. After the inhibition of RYR activity by dantrolene, LAD2 cells were stimulated by MRGPRX2 ligands c48/80 and SP. IL-13, TNF-α, CCL-1, and CCL-2 mRNA generation were perturbed after the application of RYR inhibitor (**Figure 3**). We also detected selected cytokine mRNA expression in human skin-derived MCs triggered by c48/80, and decreased IL-13, TNF-α, CCL-1, and CCL-2 mRNA were detected after dantrolene pre-treatment (**Supplementary Figure 1C**). In accordance with mRNA data, all the cytokine/chemokine detected were downregulated by RYR inhibitor at the protein level (**Figure 4**).

### 3.4 RYR3 is required in MRGPRX2-mediated LAD2 cell activation, while RYR2 has variable contribution

To further verify the role of RYR in MRGPRX2-mediated MC activation, we selectively silenced the expression of the RYR gene. Three isoforms of *RYRs* have been identified (*RYR1, RYR2*, and *RYR3*) (26). To select the RYR isoforms that potentially participate in the functional regulation of LAD2 cells, we compared the expression level of the transcripts based on the GEO dataset (GSE216269) (31). From this dataset, the expression of unstimulated LAD2 cells were selected for assessment; RYR2 and RYR3 showed relatively higher expression (**Supplementary Figure 3A**). The expression of candidate genes was further quantified by RT-qPCR in our study. In all three identified isoforms, RYR3 had the highest expression, which was followed by RYR2, and RYR1 was rarely expressed in LAD2 cells (**Supplementary Figure 3B**). Thus, we selectively knocked down the expression of RYR2 and RYR3, and both RYR2- and RYR3-selective siRNAs resulted in the effective knockdown of their respective targets (**Supplementary Figures 3C, D**).

**Figure 5:**
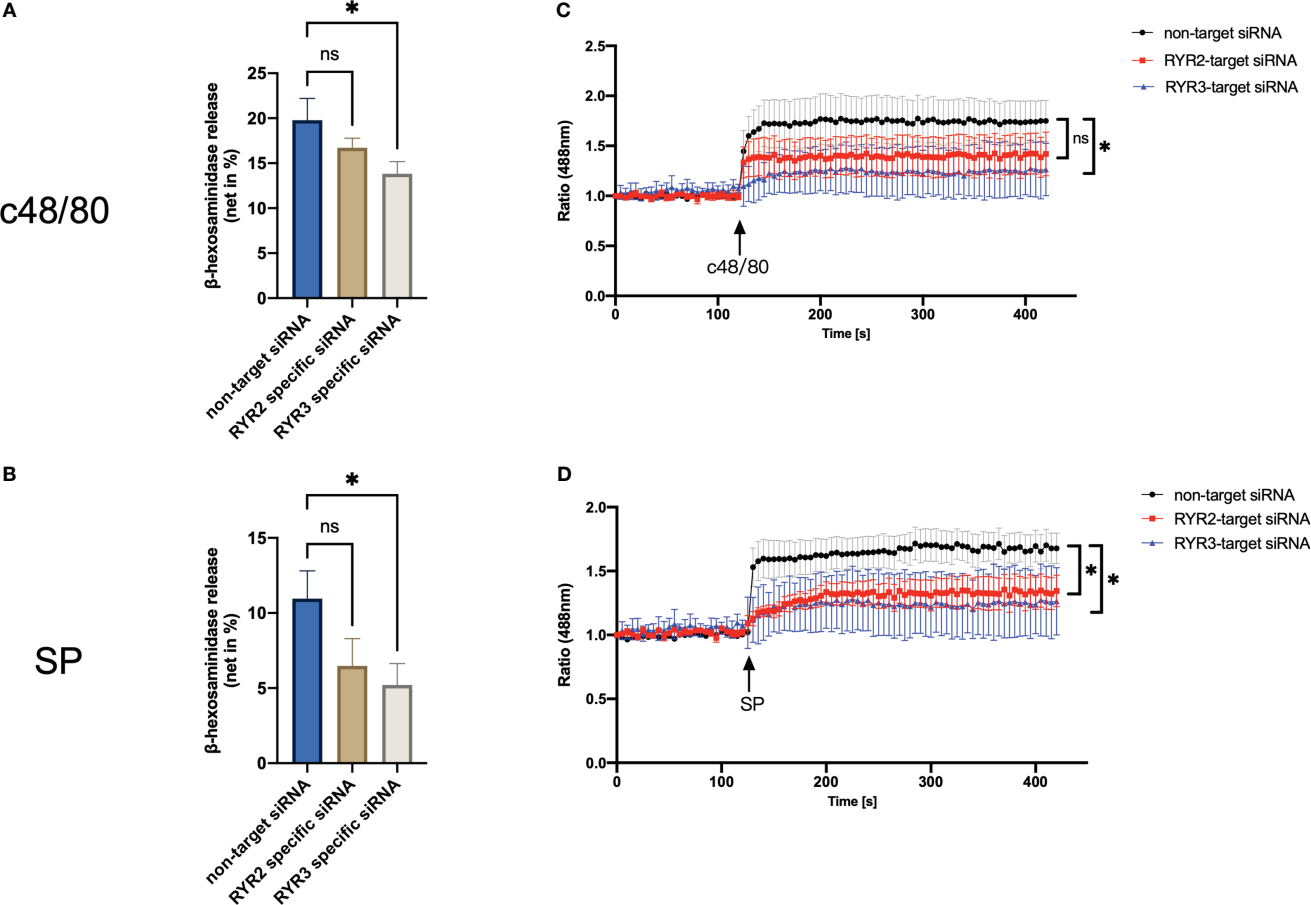
RYR3 contributes to the degranulation and calcium mobilization activated by MRGPRX2. LAD2 cells were treated with RYR2- and RYR3-specific siRNA or non-target siRNA, respectively, then **(A, B)** β‐hexosaminidase release and **(C, D)** calcium mobilization was detected. The data are from seven to nine independent experiments. ns: not significant, *p < 0.05.

Interference with RYR3 suppressed the degranulation by both agonists employed, i.e., c48/80 and SP. RYR2 siRNA also decreased the secretion of LAD2 cells, although it did not reach significance (**Figures 5A, B**). In terms of calcium mobilization, RYR3-specific siRNA decreased the calcium signal induced by both ligands (**Figures 5C, D**). RYR2 only significantly inhibited SP-triggered calcium influx; c48/80 had the tendency but did not reach significance.

**Figure 6:**
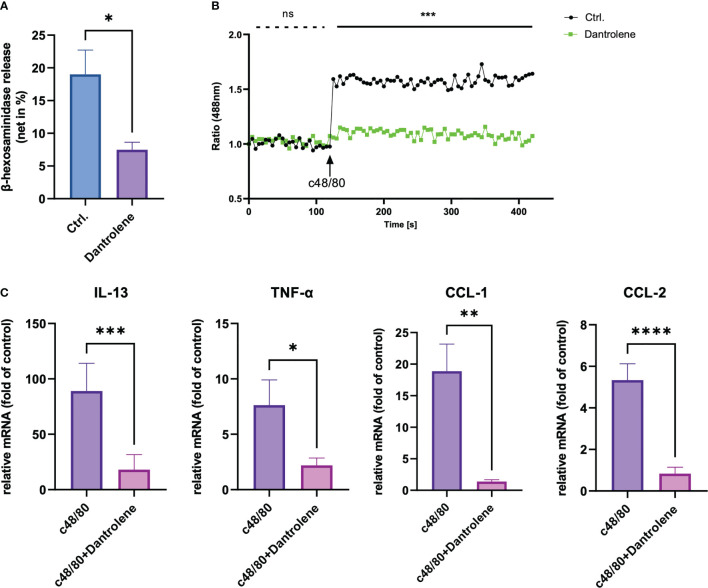
Cytokine mRNA generation *via* MRGPRX2 relies predominantly on the activation of RYR3. LAD2 cells were treated with RYR2- and RYR3-specific siRNA or non-target siRNA, then stimulated with **(A)** c48/80 (5 µg/ml) or **(B)** SP (60 µM). TNF-α, CCL-1, and CCL-2 mRNA expression were determined by RT-qPCR. The data were normalized against the cell receiving no inhibitor and stimuli. Data shown are mean ± SEM of *n* = 7. ns: not significant, *p < 0.05, **p < 0.01, ***p < 0.001, **** p<0.0001..

Then, we attempted to ascertain that MRGPRX2-mediated cytokine generation relies on RYR activation in siRNA KD LAD2 cells. RYR3 downregulated IL-13, TNF-α, CCL-1, and CCL-2 mRNA synthesis triggered by c48/80 and SP. RYR2 siRNA also downregulated all the cytokine genes detected, while only c48/80 stimulated CCL-1 and CCL-2 significantly decreased (**Figure 6**).

## 4 Discussion

MRGPRX2-mediated MC activation has drawn much attention recently, due to its non-IgE-dependent feature. Moreover, MRGPRX2-mediated MC activation is implicated in multiple dermatoses, e.g., chronic spontaneous urticaria and atopic dermatitis (1, 32, 33). In the present study, we demonstrate that RYR-sensitive calcium signaling is activated after MRGPRX2 activation. MRGPRX2-mediated MC granule secretion, calcium mobilization, and cytokine generation are inhibited by both RYR inhibitor and siRNA-mediated KD. MRGPRX2 activation is also inhibited by an RYR inhibitor in an *in vivo* mouse skin anaphylaxis model.

The induction of calcium signaling after antigen-specific IgE triggering in mast cells has been noticed for a long time. It has been recognized that the depletion of intracellular calcium stores led to the entry of extracellular calcium (34, 35). Both the activation of intracellular calcium stores and cell membrane-expressing calcium channels contribute to mast cell degranulation and cytokine production (18, 28). The classical IgE-induced calcium signal was recognized as the production of inositol 1,4,5-trisphosphate (IP3) by phospholipase Cγ (36), and the IP3/thapsigargin-sensitive pool from the endoplasmic reticulum (ER) induced the depletion of the intracellular calcium store. Depletion of this pool leads to the influx of external calcium by activating cell membrane expressing calcium channels. The calcium activation pattern was different in FcϵRI versus MRGPRX2. MRGPRX2-triggered calcium influx is quicker than that triggered by FcϵRI aggregation (14). This indicates that the calcium activation model in one channel cannot be applied to another. Considering that the intracellular calcium store is the first responder in the calcium signaling events, the ER-expressing calcium signal might be more crucial in MRGPRX2-related MC functional regulation than that in FcϵRI-mediated mast cell activation.

Intracellular calcium stores in MCs were widely believed to be activated by the IP3 receptor (IP3R) (16, 37, 38). The role of RYR, which is also an ER-expressing calcium channel, was seldom reported. We found that the calcium influx triggered by MRGPRX2 can be effectively inhibited by an RYR inhibitor, namely, dantrolene. The inhibition was consistent across two MRGPRX2 ligands applied, i.e., c48/80 and SP. Although dantrolene can effectively inhibit calcium influx activated by MRGPRX2, the inhibition was not complete. As MRGPRX2 activates multiple calcium channels, the utilization of other calcium channels also contributes to intracellular calcium oscillation, e.g., the activation of IP3R, STIM1, and Orai channels (28, 39). MC degranulation largely depends on calcium signaling, as we have previously reported with IP3R and Orai-dependent SOCE (40). Moreover, the baseline calcium level was dampened after pre-incubation with an RYR inhibitor (**Supplementary Figure 2**). Intracellular calcium level normally remains at very low concentrations, which is sufficient for the regulation of numerous basic cellular processes including proliferation, differentiation, and cellular motility (41). The decrease of intracellular calcium level at the resting state indicates that the RYR channel is involved in the maintenance of fundamental cellular function. RYR can be activated by multiple ways, including interaction with L-type calcium channel (dihydropyridine receptor) and activation of various ions (calcium and magnesium) and proteins [protein kinase A, FK506 binding proteins, calmodulin, and calcium/calmodulin-dependent protein kinase II (CaMKII)] (21). L-type calcium channel, which is a store-operated calcium channel (SOC), has been reported to mediate FcϵRI aggregation-induced MC activation (42). Moreover, MC activation by both FcϵRI aggregation and MRGPRX2 activates calcium/calmodulin (43–45).

Previous studies have demonstrated that MC degranulation is regulated by calcium signals. The role of ER-expressing IP3R, STIM1, Orai channels, and TRP channels has been profoundly illuminated in multiple studies (16, 28, 39). The receptors that cannot activate calcium influx cannot trigger degranulation, e.g., ST2/IL-33R, TLR4, or TSLPR (46–49). We found that the RYR inhibitor downregulated MRGPRX2-triggered degranulation. Partial inhibition of MRGPRX2-mediated MC degranulation by RYR inhibitor is consistent with the calcium mobilization data, indicating that the calcium signal might be compensated by other calcium channels.

It is well known that calcium signaling is essential for the generation of multiple proinflammatory cytokines (16, 50). MCs are capable of generating multiple cytokines. Based on our previous studies (51), we selected several cytokine genes to detect in the present study. CCL-1, CCL-2, TNF-α, and IL-13 mRNA and proteins were downregulated by MRGPRX2 activation. Downstream of calcium signaling, multiple signaling pathways including MAPKs and calcineurin-NFAT signaling pathways are activated (40, 52). CCL-1, CCL-2, TNF-α, and IL-13 were all regulated by TAK1, which is the kinase downstream of calmodulin (53, 54). The role of RYR in the generation of the cytokines listed was further demonstrated by the knockdown of individual RYR isoforms. Herein, we confirmed the participation of RYR3 in this process.

Finally, we used the mouse cutaneous anaphylaxis model to verify the role of RYR in Mrgprb2 activation *in vivo.* MC degranulation is associated with increased vascular permeability, which leads to vasodilation and vascular leakage. Decreased skin edema and vascular leakage triggered by SP with dantrolene pretreatment demonstrated the role of RYR in MRGPRX2-mediated skin allergy. In terms of immune cell recruitment, which represents the late-phase response following MC activation, the curtailed neutrophil recruitment represents the inhibited inflammation by RYR inhibition. Interestingly, in *ex vivo* studies, the reduction rate of β‐hexosaminidase release is less than the vascular leakage and immune cell infiltration in *in vivo* studies. Apart from the reason that to trigger a certain symptom needs adequate amount of stimulator, the release of the MC granule could be under the triggering threshold after the inhibition by dantrolene. Moreover, RYR expresses on multiple cell types, e.g., keratinocyte and neutrophils (55, 56). Pre-treatment of dantrolene can also inhibit the activation of RYR on other cells; thus, the inhibition in animal studies was higher than that in *ex vivo* experiments.

In conclusion, MRGRPX2 activation regulates pseudo-allergic reactions, and emerging compounds including FDA-approved drugs were identified as MRGPRX2 ligands. The role of MRGPRX2 is insinuated in the pathogenesis of urticaria, atopic dermatitis, and psoriasis. We identified RYR as a calcium channel, which is activated by MRGPRX2. The activation leads to MC degranulation and the generation of CCL-1, CCL-2, TNF-α, and IL-13. The *ex vivo* studies were verified by RYR isoform knockdown. Moreover, the pivotal role of RYR3 was identified. With the cutaneous anaphylaxis mouse model, we found that MRGPRX2-induced vascular permeability and immune cell recruitment were impaired by dantrolene. The present study suggests the potential novel therapeutic approaches targeting RYR for the treatment of allergic diseases.

## Data availability statement

The original contributions presented in the study are included in the article/**Supplementary Material**. Further inquiries can be directed to the corresponding authors.

## Ethics statement

The studies involving human participants were reviewed and approved by the Ethics Committee of the Second Affiliated Hospital of Xi’an Jiaotong University. The patients/participants provided their written informed consent to participate in this study. The animal study was reviewed and approved by the Ethics Committee of the Second Affiliated Hospital of Xi’an Jiaotong University.

## Author contributions

Conceptualization, ZW. Investigation, ZW, XZ, HZ, DC, XD, and DY. Data curation, ZW and XZ. Writing—original draft preparation, ZW. Writing—review and editing, ZW, SG, and WZ. Visualization, ZW. Supervision, ZW, SG, and WZ. Project administration, ZW. Funding acquisition, ZW and SG. All authors contributed to the article and approved the submitted version.
