# Supplementary material for: Activation of ryanodine-sensitive calcium store drives pseudo-allergic dermatitis via Mas-related G protein-coupled receptor X2 in mast cells
Source: Front Immunol. 2023 Jun 19;14:1207249. doi: 10.3389/fimmu.2023.1207249 (PMC10315577; doi:10.3389/fimmu.2023.1207249)
Supplement: Supplementary file 1 [file DataSheet_1.pdf]

Supplementary Material

Activation of Ryanodine Sensitive Calcium Store Drives pseudo-allergic dermatitis via Mas-Related G Protein-Coupled Receptor X2 in Mast Cells

Zhao Wang[1#], Xi Zhao[1*#], Hongmei Zhou[1], Delu Che[1], Xiaojie Du[1], Dan Ye[1], Weihui Zeng[1*], Songmei Geng[1*]

[1]Department of Dermatology, the Second Affiliated Hospital of Xi'an Jiaotong University, Xi'an, China

*** Correspondence:**
Songmei Geng: gengsongmei73@163.com

Weihui Zeng: Zengwh88@126.com

# These authors contributed equally to this work

1 Supplementary Figures

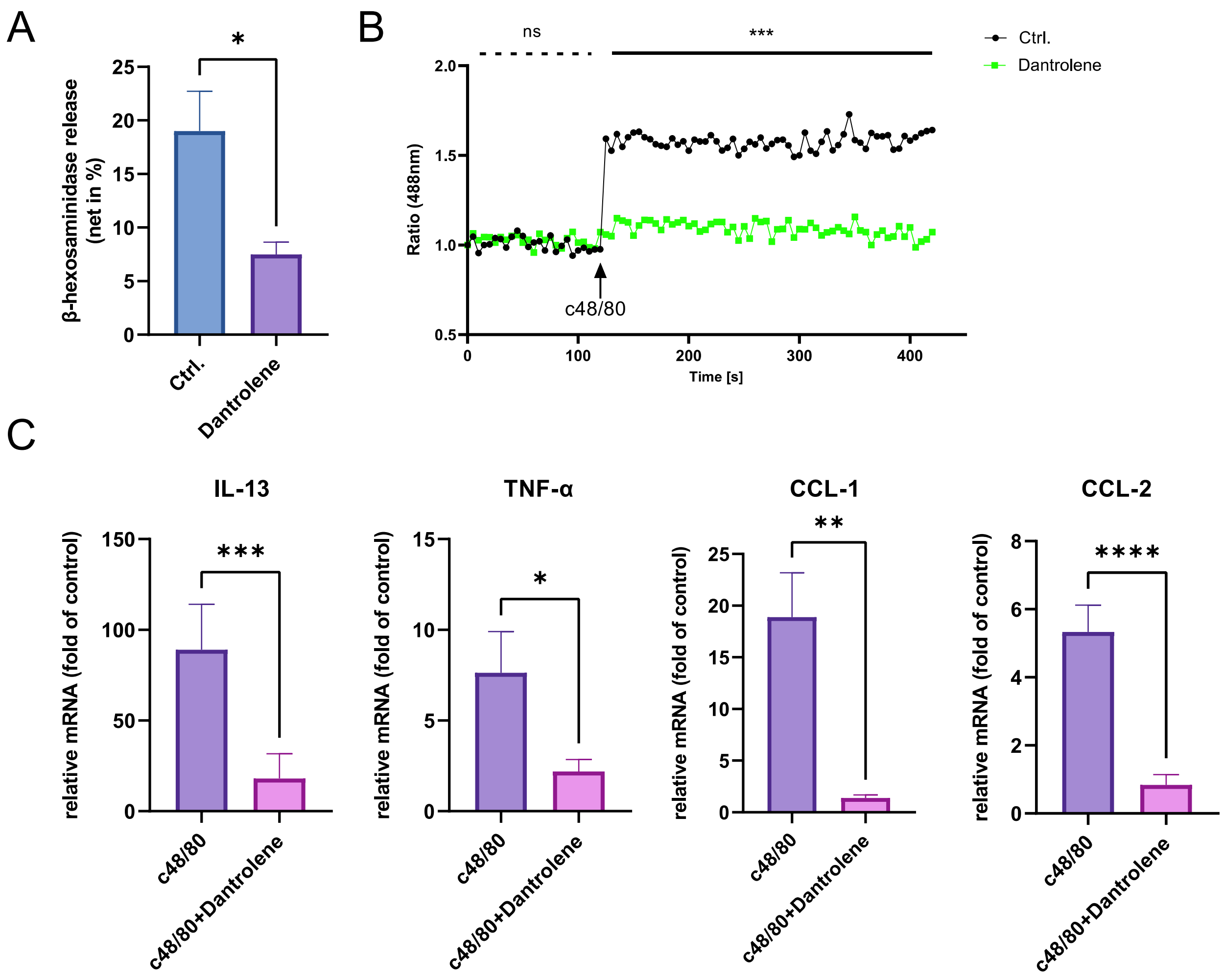

Suppl. Fig. 1. Dantrolene suppresses c48/80 induced human skin MC activation. Human skin MCs were activated by c48/80 (10 µg/ml), then (A) β-hexosaminidase release, (B) calcium mobilization and (C) mRNA expression of IL-13, TNF-alpha, CCL1 and CCL2 were determined. The data shown are mean ± SEM from 5-12 independent experiments, Ctrl.: control, inh.: inhibitor. * $p < 0.05$, ** $p < 0.01$, *** $p < 0.001$, **** $p < 0.0001$.

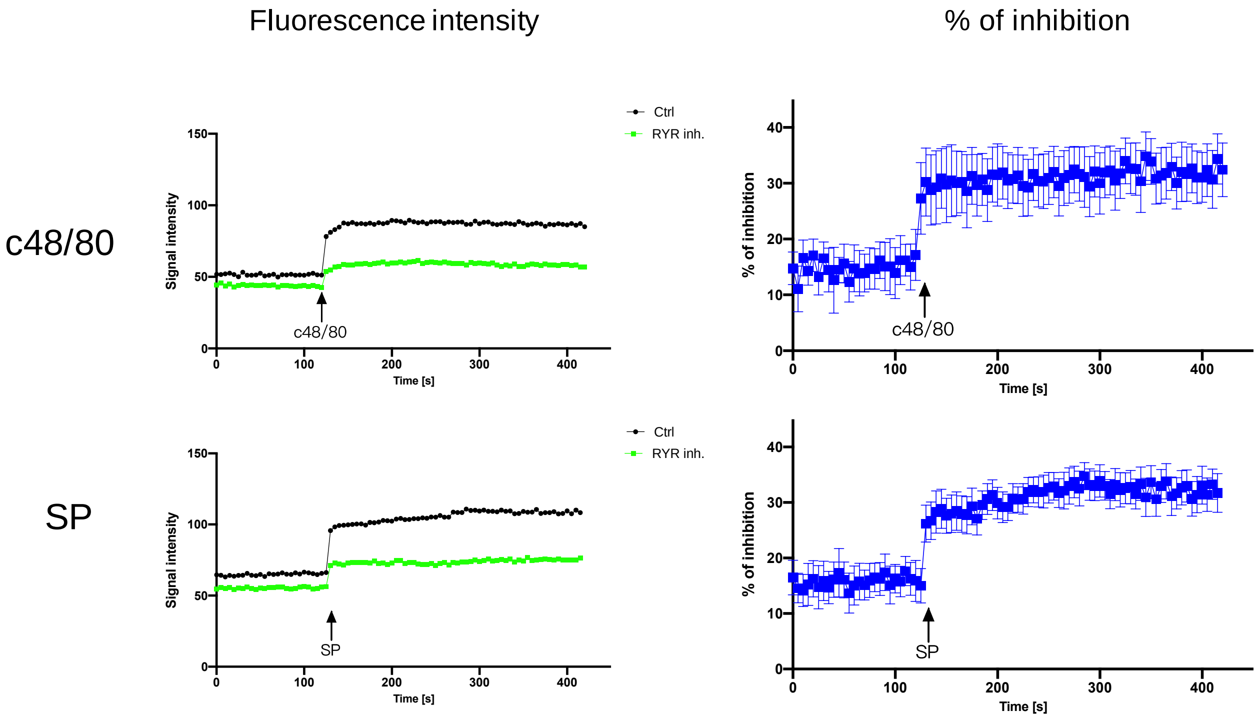

Suppl. Fig. 2. Dantrolene suppresses c48/80 and SP-induced calcium influx in LAD2 cell. LAD2 cells were treated with RYR inhibitor (dantrolene, 100 µM) for 15 min, then the cells were stimulated with c48/80 (5 µg/ml) or SP (30 µM). Calcium mobilization by recording Fluo-4 signal intensity was determined. Left panel: signal intensity, right panel: % of inhibition calculated. % of inhibition = (1-(signal intensity in Ctrl. group - signal intensity in RYR inh. group)/ signal intensity in Ctrl. Group) *100. Data are from 7-15 independent experiments. Ctrl.: control, inh.: inhibitor.

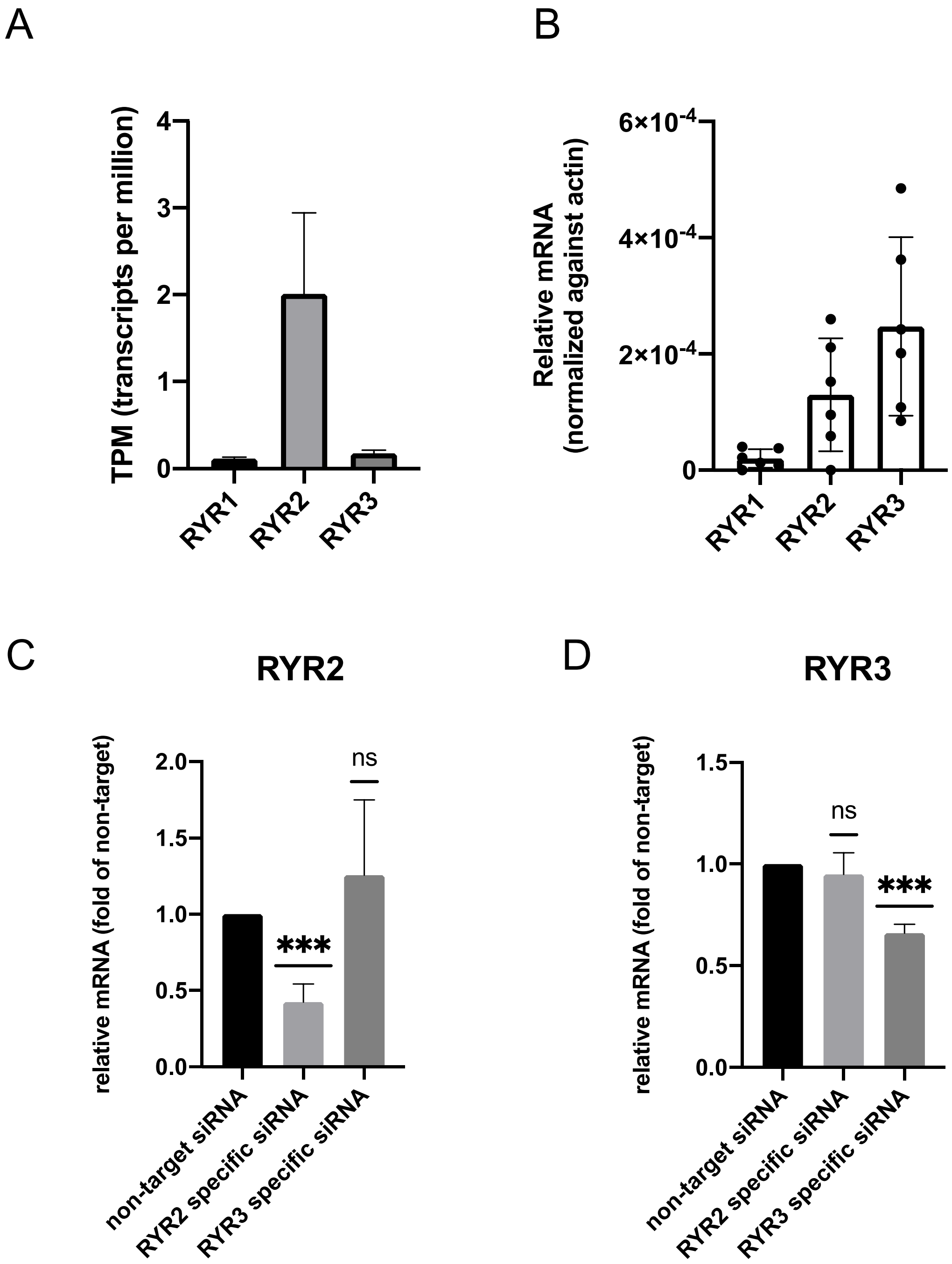

Suppl. Fig. 3. RYR2 and RYR3 express on LAD2 cells, the expression can be effectively interfered with by siRNA. (A) Expression levels (TPM = transcripts per million) of *RYR1*, *RYR2* and *RYR3* in 7 non-stimulated LAD2 samples was retrieved from the GEO database (GSE216269). (B) Expression of RYR1, RYR2 and RYR3 mRNA in LAD2 cells. C, D) LAD2 cells were treated with RYR2 and RYR3 specific siRNA, then the expression of RYR2 and RYR3 were determined by RT-qPCR. The

data was normalized against the cell receiving no inhibitor and stimuli. Data shown are mean ± SEM of n=6-7. ns: not significant, *** $p < 0.001$.
